# Evaluation of Light Chain Removal Rates in Blood Purification Therapy for Light Chain Cast Nephropathy

**DOI:** 10.1016/j.xkme.2025.100993

**Published:** 2025-03-17

**Authors:** Hisashi Kamido, Shigekazu Kurihara, Yuki Oba, Kaito Hirota, Kansei Suzuki, Masayuki Yamanouchi, Tatsuya Suwabe, Kei Kono, Kenichi Ohashi, Yoshifumi Ubara, Naoki Sawa

**Affiliations:** 1 Nephrology Center, Toranomon Hospital Branch, Kanagawa, Japan; 2 Clinical Engineering Department, Toranomon Hospital Branch, Kanagawa, Japan; 3 Nephrology Center, Toranomon Hospital, Tokyo, Japan; 4 Department of Pathology, Toranomon Hospital, Tokyo, Japan; 5 Department of Human Pathology, Graduate School of Medical and Dental Sciences, Tokyo Medical and Dental University, Tokyo, Japan

**Keywords:** Light chain cast nephropathy, multiple myeloma, online hemodiafiltration, plasma exchange

## Abstract

Acute kidney injury is a crucial prognostic factor for multiple myeloma. The most common cause is light chain cast nephropathy. The primary pathology of light chain-induced acute kidney injury involves obstruction of distal tubules due to the interaction of free light chains (FLCs) with Tamm-Horsfall protein produced there. Based on this pathology, chemotherapy is used to suppress the production of FLCs. Recently, combined blood purification therapies to remove existing FLCs have been used. However, the extent to which FLCs are removed by blood purification therapy remains unclear. We investigated the dialysis removal rates under various conditions and found that hemodialysis achieved 16% removal, plasma exchange 75%, and online hemodiafiltration varied from 20% to 31%. Although online hemodiafiltration is less effective than plasma exchange, it is a viable option that does not require albumin infusions or lead to infections. Despite hematologic remission, renal recovery was limited by a high number of casts, severe interstitial fibrosis and tubular atrophy, and the delay in treatment initiation.

## Introduction

Acute kidney injury (AKI) is a crucial prognostic factor for multiple myeloma. While the causes of AKI are diverse, light chain cast nephropathy (LCCN) is the most common.^1^ The primary pathology of light chain-induced AKI involves obstruction of distal tubules due to the interaction of free light chains (FLCs) with Tamm-Horsfall protein produced there.^1^ In addition to chemotherapy to suppress the production of FLCs, combined blood purification therapies such as plasma exchange (PE) and high-efficiency hemodialysis (HD) are used to remove existing FLCs.^2^ However, the extent to which FLCs are removed by blood purification therapy remains unclear. In this case, the removal rates of FLCs by HD, PE, and online hemodiafiltration (O-HDF) were investigated.

## Case Presentation

A 63-year-old woman with no significant past medical history was diagnosed with a compression fracture 3 weeks prior. She was referred to our hospital due to a decline in kidney function, which was noted following a decrease in urine output 1 week before her admission.

On admission, her height was 156 cm, weight was 50 kg, body mass index was 20.5 kg/m^2^, blood pressure was 141/86 mmHg, heart rate was 100 bpm, respiratory rate was 30 breaths per minute, and SpO_2_ was 98% on room air. Physical examination revealed anemia in the palpebral conjunctiva.

Laboratory results showed the following: white blood cell count, 10,400/μL; hemoglobin, 6.1 g/dL; platelet count, 161 × 10^4^/mL; lactate dehydrogenase, 287 U/L; albumin, 3.9 g/dL; urea nitrogen, 153 mg/dL; creatinine, 25.5 mg/dL; estimated glomerular filtration rate, 1.3 mL/min/1.73 m^2^; sodium, 132 mmol/L; potassium, 6.2 mmol/L; chloride, 91 mmol/L; albumin-adjusted calcium, 10.2 mg/dL; C-reactive protein, 0.3 mg/dL; immunoglobulin (Ig)G, 251 mg/dL; IgA, 5.3 mg/dL; IgM, 4.4 mg/dL; β2-microglobulin, 52 mg/L; bicarbonate, 7 mmol/L; κ light chain, 20 mg/L; λ light chain, 47,161 mg/L; and positive monoclonal protein λ. Urinalysis showed: occult blood, negative; proteinuria, 3+; red blood cells, <1/high-power field; urinary protein excretion, 7.6 g/g creatinine; N-acetyl-β-D-glucosaminidase, 22 U/L; β2-microglobulin, 20 mg/L; and Bence Jones protein λ, positive (Table 1).

**Table 1:** Laboratory Data on Admission

Laboratory test	Result	Normal range
Urinalysis		
Protein	3+	Negative
Occult blood	Negative	Negative
Erythrocytes (/HPF)	<1	<5
Leukocytes (/HPF)	1-4	<5
Protein creatinine ratio (g/gCr)	7.6	<0.15
β2-microglobulin (mg/L)	20.1	<0.2
N-acetyl-β-D-glucosaminidase (U/L)	22.0	<11.5
Bence Jones proteinuria	BJP-λ	Negative
Blood tests		
White blood cells (× 10^3^/μL)	10.4	3.3-8.6
Plasma cells (%)	6.5	Negative
Hemoglobin (g/dL)	6.1	11.6-14.8
Platelet (× 10^3^/μL)	161	158-348
Creatine kinase (U/L)	135	41-153
Aspartate aminotransferase (U/L)	11	13-30
Alanine aminotransferase (U/L)	12	7-23
Lactate dehydrogenase (U/L)	287	124-222
Alkaline phosphatase (U/L)	81	38-113
γ-glutamyl transferase (U/L)	19	9-32
Total bilirubin (mg/dL)	0	0.4-1.5
Total protein (g/dL)	7.0	6.6-8.1
Albumin (g/dL)	3.9	4.1-5.1
Low-density lipoprotein (mg/dL)	81	65-139
Urea nitrogen (mg/dL)	153	8-20
Creatinine (mg/dL)	25.5	0.46-0.79
Estimated glomerular filtration rate (mL/min/1.73 m^2^)	1.3	>90
Sodium (mEq/L)	132	138-145
Potassium (mEq/L)	6.2	3.6-4.8
Chloride (mEq/L)	91	101-108
Calcium (mg/dL)	10.1	8.8-10.1
Phosphate (mg/dL)	12.7	2.7-4.6
C-reactive protein (mg/dL)	0.3	<0.14
Immunoglobulin G (mg/dL)	251	861-1747
Immunoglobulin A (mg/dL)	5.3	93-393
Immunoglobulin M (mg/dL)	4.4	50-269
M protein	BJP-λ	Negative
κ light chain (mg/L)	20	3.3-19.4
λ light chain (mg/L)	47,161	5.7-26.3
κ/λ ratio	<0.01	0.26-1.65
β2-microglobulin (mg/L)	52	0.5-2.0
Ferritin (μg/L)	1,593	5-120

Abbreviations: gCr, grams creatinine; HPF, high-power field.

**Figure 1:**
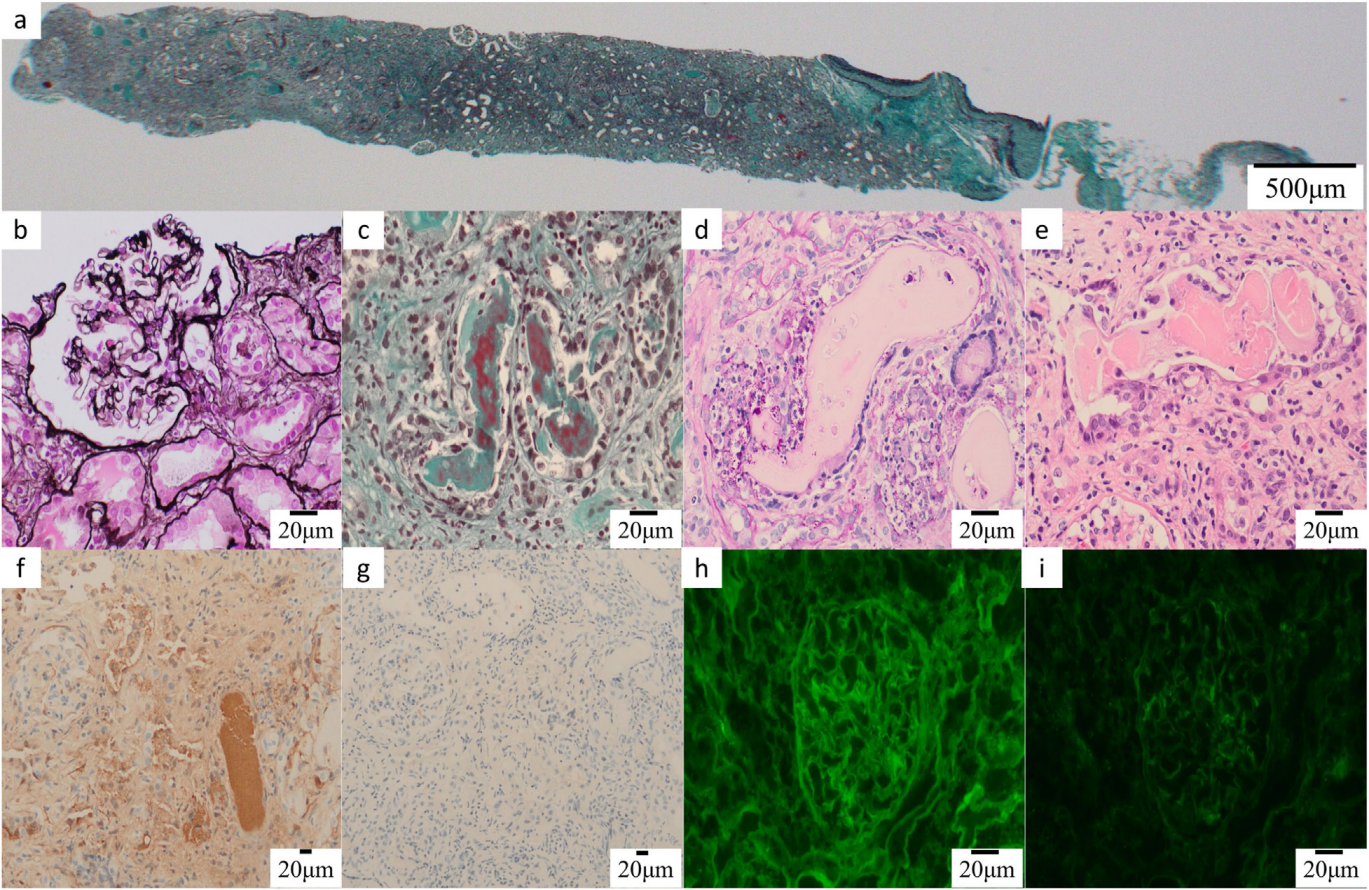
Kidney biopsy. (a) Greater than 90% of the tubular interstitium in the cortical region exhibits fibrosis and inflammatory cell infiltration by (Masson’s trichome staining). (b) The glomeruli are intact, but the proximal tubular epithelial cells are damaged (periodic acid methenamine silver staining). (c) Cast formation is stained red and blue by Masson’s trichrome staining. (d) Cast formation is not visible by periodic acid–Schiff. (e) Cast formation is stained red by hematoxylin and eosin. (f) Immunohistological staining for λ light chain shows it is abundant throughout the tubules, especially in the cast lesion of the distal tubule. (g) Immunohistological staining for κ light chain is negative. (h) Immunofluorescent staining shows λ light chain is present along the glomeruli, the proximal tubule, and the distal tubules. (i) Immunofluorescent staining for κ light chain is negative.

Radiograph of the skull revealed multiple punched-out lesions, and computed tomography revealed multiple osteolytic lesions in the cervical spine and pelvis, along with compression fractures at Th11 and L1-L2. Bone marrow biopsy demonstrated 89.6% plasma cells, with flow cytometry showing 74.7% of the cells were CD38-positive and cy λ-positive. No chromosomal abnormalities were detected. A kidney biopsy obtained 21 glomeruli without global sclerosis or crescents. Although the glomeruli appeared intact, interstitial fibrosis and tubular atrophy were severe, especially in the distal tubules, which exhibited prominent cast formation (Fig 1a-e). Inflammatory cell infiltration was noted in and around the casts.

Immunohistochemical analysis revealed stronger λ light chain positivity throughout the tubules, particularly in the cast lesions of the distal tubules (Fig 1f), compared to κ light chains (Fig 1g). Similarly, immunofluorescence staining showed granular λ light chain deposition along the glomeruli, proximal tubules, and distal tubules (Fig 1h), with no corresponding staining for κ light chains (Fig 1i). Congo red staining was negative, and electron microscopy did not identify any electron-dense deposits.

Based on the above findings, the patient was diagnosed with AKI due to LCCN associated with symptomatic multiple myeloma. Treatment was initiated with a combination chemotherapy with daratumumab, bortezomib, and dexamethasone and blood purification therapy. Blood purification therapy was primarily conducted 3 times a week. Gradually, the efficiency of dialysis was improved, with careful attention given to preventing dialysis disequilibrium syndrome. The λ light chain levels were measured before and after treatments of HD, PE, and 3 different patterns of O-HDF (urea nitrogen levels were not measured). In HD (dialyzer ABH-22PA (Asahi Kasei Medical Co, Ltd), blood flow 120 mL/min, dialysate flow 600 mL/min, duration 4 hours), the λ level decreased from 34,576 mg/dL to 29,048 mg/dL, with a removal rate of 16%. In PE (fresh frozen plasma 30U), the λ level decreased from 32,647 mg/dL to 8,185 mg/dL, achieving a 75% removal rate. With O-HDF pattern 1 (blood flow 120 mL/min, dialysate flow 700 mL/min, substitution volume 20 L, duration 4 hours), λ decreased from 23,711 mg/dL to 18,496 mg/dL, with a 22% removal rate. With O-HDF pattern 2 (blood flow 300 mL/min, dialysate flow 700 mL/min, substitution volume 20 L, duration 4 hours), λ decreased from 12,251 mg/dL to 9,779 mg/dL, with a 20% removal rate. With O-HDF pattern 3 (blood flow 300 mL/min, dialysate flow 700 mL/min, substitution volume 50 L, duration 5 hours), λ decreased from 4,954 mg/dL to 3,394 mg/dL, with a 31% removal rate (Table 2).

**Table 2:** Light Chain Removal Rates in Blood Purification Therapy

Modalities	Dialyzer	Blood Flow (mL/min)	Dialysate Flow (mL/min)	Substitution Volume (L, Predilution)	Duration (h)	Pre λ (mg/dL)	Post λ (mg/dL)	Removal Efficiency (%)
HD	APS22PA	120	600	—	4	34,576	29,048	16
PE	—	—	—	FFP 30U		32,647	8,185	75
O-HDF 1	APH22PA	120	700	20	4	23,711	18,496	22
O-HDF 2	APH22PA	300	700	20	4	12,251	9,779	20
O-HDF 3	APH22PA	300	700	50	5	4,954	3,394	31

*Note:* Blood purification therapy was primarily conducted 3 times a week. Gradually, the efficiency of dialysis was improved, with careful attention given to preventing dialysis disequilibrium syndrome. The λ light chain levels were measured before and after treatments with HD, PE, and 3 different patterns of O-HDF.

Abbreviations: FFP, fresh frozen plasma; HD, hemodialysis; O-HDF, online hemodiafiltration; PE, plasma exchange.

**Figure 2:**
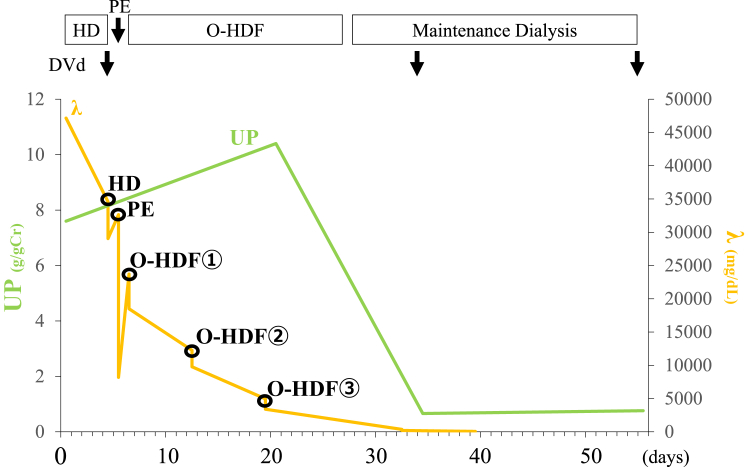
Clinical course from admission and after treatment. Despite a significant decrease in free light chains (λ) and achieving hematological remission, renal function did not improve. Consequently, blood purification therapy was discontinued, and the patient transitioned to maintenance dialysis. Abbreviations: DVd, daratumumab, bortezomib, and dexamethasone; HD, hemodialysis; O-HDF, online hemodiafiltration; PE, plasma exchange; UP, urinary protein-to-creatinine ratio.

Despite a significant decrease in FLC (λ) and achieving hematological remission, kidney function did not improve due to severe interstitial fibrosis and tubular atrophy observed in renal pathology. Consequently, blood purification therapy was discontinued, and the patient transitioned to maintenance dialysis (Fig 2).

## Discussion

In this case, we evaluated the removal rates of FLCs in blood purification therapy for LCCN. While the causes of AKI in multiple myeloma are diverse,^1^ the effectiveness of blood purification therapy in removing FLCs has only been demonstrated in LCCN.^2^ Therefore, it is crucial to first clarify the pathology of AKI using kidney biopsy.

For blood purification therapy, PE and high-efficiency HD using a high cut-off (45-60 kDa) dialyzer are primarily used. Previous studies have shown significant results for PE. Leung et al^3^ reported that after a median of 6 PE sessions, 75% of patients who achieved a reduction in FLCs of >50% experienced improved renal outcomes at 2 months. Burnette et al^4^ reported improved renal outcomes in 86% of patients 6 months after a median of 8 PE sessions. Regarding high cut-off dialysis for LCCN, 2 randomized controlled trials, the MYRE study and the EuLITE study, have been conducted.^5^^,^^6^ The MYRE study^5^ showed that 8 high cut-off hemodialysis sessions over 10 days significantly improved kidney function at 6 and 12 months compared to conventional HD. However, the EuLITE study^6^ did not achieve significant renal recovery at 90 days and had a higher rate of infectious complications, with worse 2-year survival rates.

Previous reports have shown that O-HDF is more effective than HD in removing FLCs in multiple myeloma, although only 1 case of LCCN was included.^7^ In the present study, we investigated dialysis removal rates under various conditions and found that O-HDF achieved about half the dialysis removal rate of PE. While high cut-off hemodialysis required substantial albumin supplementation due to frequent dialysis,^5^^,^^6^ O-HDF did not necessitate albumin infusions or result in infections.

Despite achieving hematologic remission after 1 cycle of chemotherapy, kidney function did not recover in this case. Contributing factors include the delay in treatment initiation (3 months after symptom onset). Previous reports have indicated that patients who successfully wean off dialysis typically receive treatment within 5 days to 3 weeks of symptom onset.^8^ Furthermore, renal pathology indicators such as the number of casts and degree of interstitial fibrosis and tubular atrophy are crucial for predicting renal prognosis.^9^ This case showed a high number of casts and severe interstitial fibrosis and tubular atrophy, suggesting that kidney damage was already advanced by the time treatment began, which likely reduced the effectiveness of light chain removal therapies.

Combining blood purification therapies is crucial for LCCN. HF has the highest FLC removal rate, but O-HDF can achieve about half the rate of HF without reducing albumin levels or resulting in infections. However, severe renal pathology and delayed treatment necessitate a decision to discontinue such therapies due to limited effectiveness.
